# Supplementary material for: Mortality and morbidity of asthma and chronic obstructive pulmonary disease associated with ambient environment in metropolitans in Taiwan
Source: PLoS One. 2021 Jul 6;16(7):e0253814. doi: 10.1371/journal.pone.0253814 (PMC8259956; doi:10.1371/journal.pone.0253814)

**S1 File**

S1 Table. Meta-analyses estimated cumulative 26-day relative risk (RR) and 95% confidence interval (CI) of deaths from asthma and COPD associated with the extreme temperatures in metropolitan Cities in Taiwan

|  | |  | |  | Tavg. at 5th percentile  (14.1°C) | | Tavg. at 99th percentile  (31.3°C) | |
| --- | --- | --- | --- | --- | --- | --- | --- | --- |
| **Diseases** | **Sex** | | **Age** | | **RR** | **95% CI** | **RR** | **95% CI** |
| Asthma | Male | | >65 | | 1.19 | 0.41 - 3.45 | 0.88 | 0.30 - 2.60 |
|  | Female | | >65 | | 0.34 | 0.09 - 1.28 | 1.61 | 0.28 - 9.27 |
| COPD | Male | | >65 | | 1.38 | 1.05 - 1.80 | 1.22 | 0.84 - 1.75 |
|  | Female | | >65 | | 1.07 | 0.58 - 1.96 | 0.99 | 0.50 - 1.94 |

S2 Table. Meta-analyses estimated cumulative 4-day relative risk (RR) and 95% confidence interval (CI) of emergency room visits for asthma and COPD by sex and age associated with the extreme temperatures in metropolitan Cities in Taiwan

|  | |  | |  | Tavg. at 5th percentile  (14.1°C) | | Tavg. at 99th percentile  (31.3°C) | |
| --- | --- | --- | --- | --- | --- | --- | --- | --- |
| **Diseases** | **Sex** | | **Age** | | **RR** | **95% CI** | **RR** | **95% CI** |
| Asthma | Male | | 0-18 | | 1.00 | 0.98 - 1.01 | 0.93 | 0.81 - 1.06 |
|  |  | | 19-39 | | 0.98 | 0.96 - 0.99 | 1.02 | 0.93 - 1.12 |
|  |  | | 40-64 | | 0.97 | 0.96 - 0.98 | 1.15 | 1.05 - 1.27 |
|  |  | | >65 | | 0.96 | 0.95 - 0.97 | 1.16 | 1.06 - 1.27 |
|  | Female | | 0-18 | | 1.00 | 0.99 - 1.01 | 0.96 | 0.85 - 1.09 |
|  |  | | 19-39 | | 0.97 | 0.96 - 0.98 | 1.14 | 1.02 - 1.26 |
|  |  | | 40-64 | | 0.96 | 0.95 - 0.98 | 1.17 | 1.01 - 1.34 |
|  |  | | >65 | | 0.98 | 0.97 - 1.00 | 0.96 | 0.87 - 1.06 |
| COPD | Male | | 40-64 | | 0.99 | 0.96 - 1.01 | 0.96 | 0.80 - 1.16 |
|  |  | | >65 | | 0.99 | 0.97 - 1.00 | 0.99 | 0.91 - 1.08 |
|  | Female | | 40-64 | | 0.97 | 0.93 - 1.01 | 1.16 | 0.81 - 1.67 |
|  |  | | >65 | | 1.00 | 0.98 - 1.03 | 1.03 | 0.88 - 1.20 |

S3 Table. Meta-analyses estimated cumulative 8-day relative risk (RR) and 95% confidence interval (CI) of outpatient visits for asthma and COPD by sex and age associated with the extreme temperatures in metropolitan Cities in Taiwan

|  | |  | |  | Tavg. at 5th percentile  (14.1°C) | | Tavg. at 99th percentile  (31.3°C) | |
| --- | --- | --- | --- | --- | --- | --- | --- | --- |
| **Diseases** | **Sex** | | **Age** | | **RR** | **95% CI** | **RR** | **95% CI** |
| Asthma | Male | | 0-18 | | 1.34 | 1.22 - 1.47 | 1.02 | 0.97 - 1.07 |
|  |  | | 19-39 | | 0.90 | 0.73 - 1.12 | 1.01 | 0.98 - 1.03 |
|  |  | | 40-64 | | 0.83 | 0.70 - 0.99 | 1.01 | 0.99 - 1.04 |
|  |  | | >65 | | 0.90 | 0.79 - 1.02 | 1.01 | 0.97 - 1.06 |
|  | Female | | 0-18 | | 1.54 | 1.47 - 1.60 | 1.00 | 0.96 - 1.03 |
|  |  | | 19-39 | | 0.99 | 0.85 - 1.14 | 1.03 | 0.98 - 1.08 |
|  |  | | 40-64 | | 0.83 | 0.68 - 1.01 | 1.02 | 0.99 - 1.05 |
|  |  | | >65 | | 0.89 | 0.80 - 0.99 | 1.00 | 0.97 - 1.04 |
| COPD | Male | | 40-64 | | 0.98 | 0.97 – 1.00 | 0.99 | 0.93 - 1.06 |
|  |  | | >65 | | 1.00 | 0.98 - 1.01 | 0.98 | 0.95 - 1.02 |
|  | Female | | 40-64 | | 0.99 | 0.96 - 1.02 | 0.99 | 0.89 - 1.10 |
|  |  | | >65 | | 0.98 | 0.95 – 1.00 | 0.99 | 0.94 - 1.05 |

S4 Table. Meta-analyses estimated cumulative 26-day relative risk (RR) and 95% confidence interval (CI) of deaths from asthma and COPD for the elderly men and women associated with daily 90th percentile PM_2.5_ (55 μg/m^3^) relative to Q1 level (17μg/m^3^)

|  | |  | |  | Model without adjusting temperature | | Model adjusted temperature | |
| --- | --- | --- | --- | --- | --- | --- | --- | --- |
| **Diseases** | **Sex** | | **Age** | | **RR** | **95% CI** | **RR** | **95% CI** |
| Asthma | Male | | >65 | | - | - | - | - |
|  | Female | | >65 | | - | - | - | - |
| COPD | Male | | >65 | | 0.99 | 0.89 - 1.10 | 0.97 | 0.86 - 1.08 |
|  | Female | | >65 | | - | - | - | - |

S5 Table. Meta-analyses estimated cumulative 6-day relative risk (RR) and 95% confidence interval (CI) of emergency room visits for asthma and COPD associated with daily 90th percentile PM_2.5_ (55 μg/m^3^) relative to Q1 levels (17μg/m^3^) by sex and age

|  | |  | |  | Model without adjusting temperature | | Model adjusted temperature | |
| --- | --- | --- | --- | --- | --- | --- | --- | --- |
| Diseases | Sex | | Age | | RR | 95% CI | RR | 95% CI |
| Asthma | Male | | 0-18 | | 1.04 | 1.00 - 1.08 | 1.04 | 1.01 - 1.08 |
|  |  | | 19-39 | | 1.02 | 0.98 - 1.06 | 1.02 | 0.98 - 1.06 |
|  |  | | 40-64 | | 1.04 | 0.97 - 1.09 | 1.03 | 0.97 - 1.09 |
|  |  | | >65 | | 1.05 | 0.98 - 1.09 | 1.04 | 0.98 - 1.09 |
|  | Female | | 0-18 | | 1.06 | 0.99 - 1.14 | 1.07 | 1.00 - 1.14 |
|  |  | | 19-39 | | 1.06 | 1.02 - 1.10 | 1.05 | 1.01 - 1.08 |
|  |  | | 40-64 | | 1.07 | 1.02 - 1.12 | 1.05 | 1.00 - 1.10 |
|  |  | | >65 | | 0.99 | 0.95 - 1.04 | 0.99 | 0.94 - 1.03 |
| COPD | Male | | 40-64 | | 1.04 | 0.95 - 1.14 | 1.05 | 0.96 - 1.16 |
|  |  | | >65 | | 1.06 | 1.02 - 1.09 | 1.06 | 1.03 - 1.10 |
|  | Female | | 40-64 | | 1.08 | 0.94 - 1.24 | 1.04 | 0.88 - 1.24 |
|  |  | | >65 | | 1.07 | 1.01 - 1.14 | 1.07 | 1.00 - 1.15 |

S6 Table. Meta-analyses estimated cause-specific cumulative 6-day relative risk (RR) and 95% confidence interval (CI) of outpatient visits for asthma and COPD associated with daily 90th percentile PM_2.5_ (55 μg/m^3^) relative to Q1 levels (17μg/m^3^) by sex and age

|  | |  | |  | Model without adjusting temperature | | Model adjusted temperature | |
| --- | --- | --- | --- | --- | --- | --- | --- | --- |
| Diseases | Sex | | Age | | RR | 95% CI | RR | 95% CI |
| Asthma | Male | | 0-18 | | 1.15 | 1.07 - 1.24 | 1.15 | 1.09 - 1.22 |
|  |  | | 19-39 | | 1.08 | 1.01 - 1.14 | 1.06 | 1.01 - 1.11 |
|  |  | | 40-64 | | 1.04 | 1.02 - 1.06 | 1.02 | 0.98 - 1.06 |
|  |  | | >65 | | 1.04 | 0.99 - 1.08 | 1.02 | 0.99 - 1.06 |
|  | Female | | 0-18 | | 1.11 | 1.06 - 1.17 | 1.12 | 1.08 - 1.15 |
|  |  | | 19-39 | | 1.08 | 1.05 - 1.12 | 1.06 | 1.04 - 1.08 |
|  |  | | 40-64 | | 1.05 | 1.01 - 1.09 | 1.02 | 0.99 - 1.06 |
|  |  | | >65 | | 1.03 | 0.99 - 1.07 | 1.01 | 0.96 - 1.06 |
| COPD | Male | | 40-64 | | 1.00 | 0.97 - 1.03 | 0.99 | 0.96 - 1.02 |
|  |  | | >65 | | 0.99 | 0.97 - 1.01 | 0.99 | 0.97 – 1.00 |
|  | Female | | 40-64 | | 1.00 | 0.94 - 1.06 | 0.99 | 0.93 - 1.06 |
|  |  | | >65 | | 1.00 | 0.98 - 1.02 | 1.00 | 0.97 - 1.02 |

S7 Table. Meta-analyses estimated cumulative 6-day relative risk (RR) and 95% confidence interval (CI) of deaths from asthma and COPD associated with daily 90th percentile ozone (43 ppb) relative to Q1 levels (20.8 ppb) for the elderly by sex

|  | |  | |  | Model without adjusting temperature | | Model adjusted temperature | |
| --- | --- | --- | --- | --- | --- | --- | --- | --- |
| **Diseases** | **Sex** | | **Age** | | **RR** | **95% CI** | **RR** | **95% CI** |
| Asthma | Male | | >65 | | 0.90 | 0.66 - 1.23 | 0.85 | 0.62 - 1.16 |
|  | Female | | >65 | | 0.95 | 0.73 - 1.24 | 0.93 | 0.72 - 1.20 |
| COPD | Male | | >65 | | 0.97 | 0.90 - 1.05 | 0.95 | 0.89 - 1.03 |
|  | Female | | >65 | | 1.06 | 0.95 - 1.18 | 1.09 | 0.97 - 1.22 |

S8 Table. Meta-analyses estimated cause-specific cumulative 6-day relative risk (RR) and 95% confidence interval (CI) of emergency room visits for asthma and COPD associated with daily 90th percentile ozone (43 ppb) relative to Q1 levels (20.8 ppb) by sex and age

|  | |  | |  | Model without adjusting temperature | | Model adjusted temperature | |
| --- | --- | --- | --- | --- | --- | --- | --- | --- |
| Diseases | Sex | | Age | | RR | 95% CI | RR | 95% CI |
| Asthma | Male | | 0-18 | | 1.15 | 1.05 - 1.27 | 1.15 | 1.03 - 1.29 |
|  |  | | 19-39 | | 1.15 | 1.07 - 1.24 | 1.16 | 1.07 - 1.25 |
|  |  | | 40-64 | | 1.10 | 1.00 - 1.20 | 1.11 | 1.01 - 1.23 |
|  |  | | >65 | | 1.14 | 1.05 - 1.25 | 1.15 | 1.05 - 1.25 |
|  | Female | | 0-18 | | 1.18 | 1.02 - 1.37 | 1.18 | 1.01 - 1.39 |
|  |  | | 19-39 | | 1.15 | 1.02 - 1.29 | 1.16 | 1.04 - 1.31 |
|  |  | | 40-64 | | 1.19 | 1.03 - 1.36 | 1.21 | 1.05 - 1.39 |
|  |  | | >65 | | 1.11 | 1.02 - 1.22 | 1.11 | 1.01 - 1.21 |
| COPD | Male | | 40-64 | | 1.13 | 1.00 - 1.28 | 1.13 | 0.97 - 1.32 |
|  |  | | >65 | | 1.14 | 1.04 - 1.24 | 1.12 | 1.03 - 1.23 |
|  | Female | | 40-64 | | 1.42 | 0.98 - 2.05 | 1.38 | 0.92 - 2.05 |
|  |  | | >65 | | 1.10 | 0.94 - 1.29 | 1.09 | 0.93 - 1.27 |

S9 Table. Meta-analyses estimated cause-specific cumulative 6-day relative risk (RR) and 95% confidence interval (CI) of outpatient visits for asthma and COPD associated with daily 90th percentile ozone (43 ppb) relative to Q1 levels (21 ppb) by sex and age

|  | |  | |  | Model without adjusting temperature | | Model adjusted temperature | |
| --- | --- | --- | --- | --- | --- | --- | --- | --- |
| Diseases | Sex | | Age | | RR | 95% CI | RR | 95% CI |
| Asthma | Male | | 0-18 | | 1.17 | 1.10 - 1.25 | 1.13 | 1.05 - 1.21 |
|  |  | | 19-39 | | 1.10 | 1.05 - 1.14 | 1.07 | 1.04 - 1.10 |
|  |  | | 40-64 | | 1.05 | 0.97 - 1.13 | 1.03 | 0.98 - 1.09 |
|  |  | | >65 | | 1.02 | 0.96 - 1.07 | 1.00 | 0.98 - 1.02 |
|  | Female | | 0-18 | | 1.14 | 1.09 - 1.20 | 1.10 | 1.04 - 1.15 |
|  |  | | 19-39 | | 1.13 | 1.08 - 1.17 | 1.10 | 1.06 - 1.14 |
|  |  | | 40-64 | | 1.06 | 1.02 - 1.10 | 1.05 | 1.02 - 1.08 |
|  |  | | >65 | | 1.02 | 0.95 - 1.11 | 1.01 | 0.96 - 1.06 |
| COPD | Male | | 40-64 | | 0.99 | 0.93 - 1.05 | 0.97 | 0.91 - 1.04 |
|  |  | | >65 | | 0.99 | 0.97 - 1.02 | 0.97 | 0.95 - 1.00 |
|  | Female | | 40-64 | | 1.01 | 0.95 - 1.08 | 1.00 | 0.93 - 1.08 |
|  |  | | >65 | | 1.03 | 0.98 - 1.07 | 1.00 | 0.96 - 1.05 |

S1 Fig. Daily cause-specific deaths, ERVs, and outpatient visits by sex and age


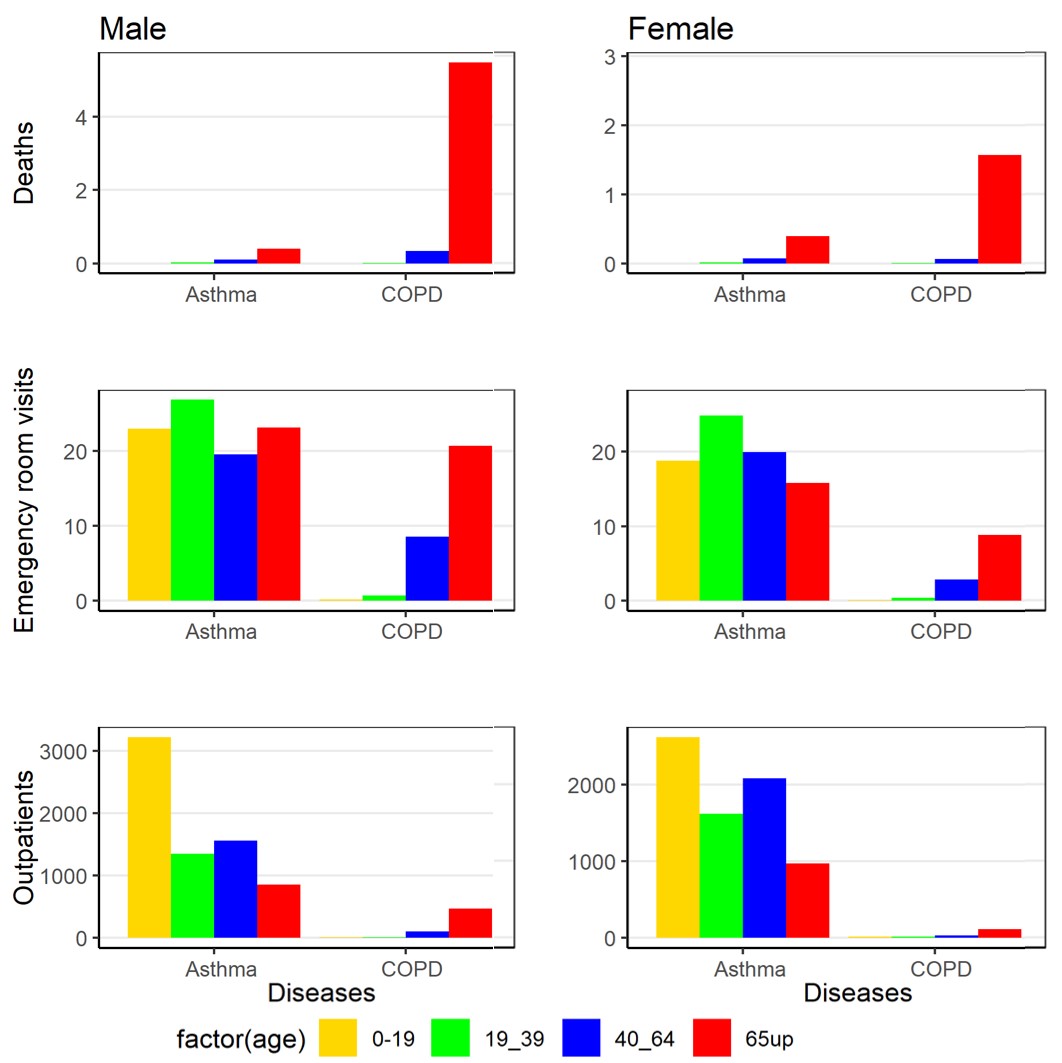


S2 Fig. Trends of environmental conditions from 2005 to 2016 in metropolitan Cities in Taiwan


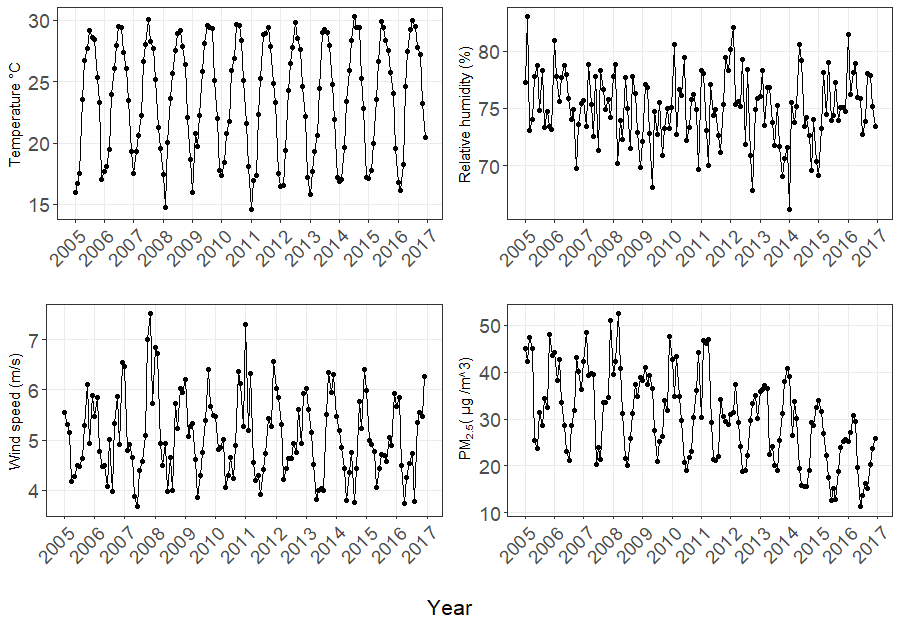


S3 Fig. Cumulative 26-day (lag 0-25) relative risk (95% confidence interval) of deaths from COPD in 40-64 years old men associated with daily average temperature


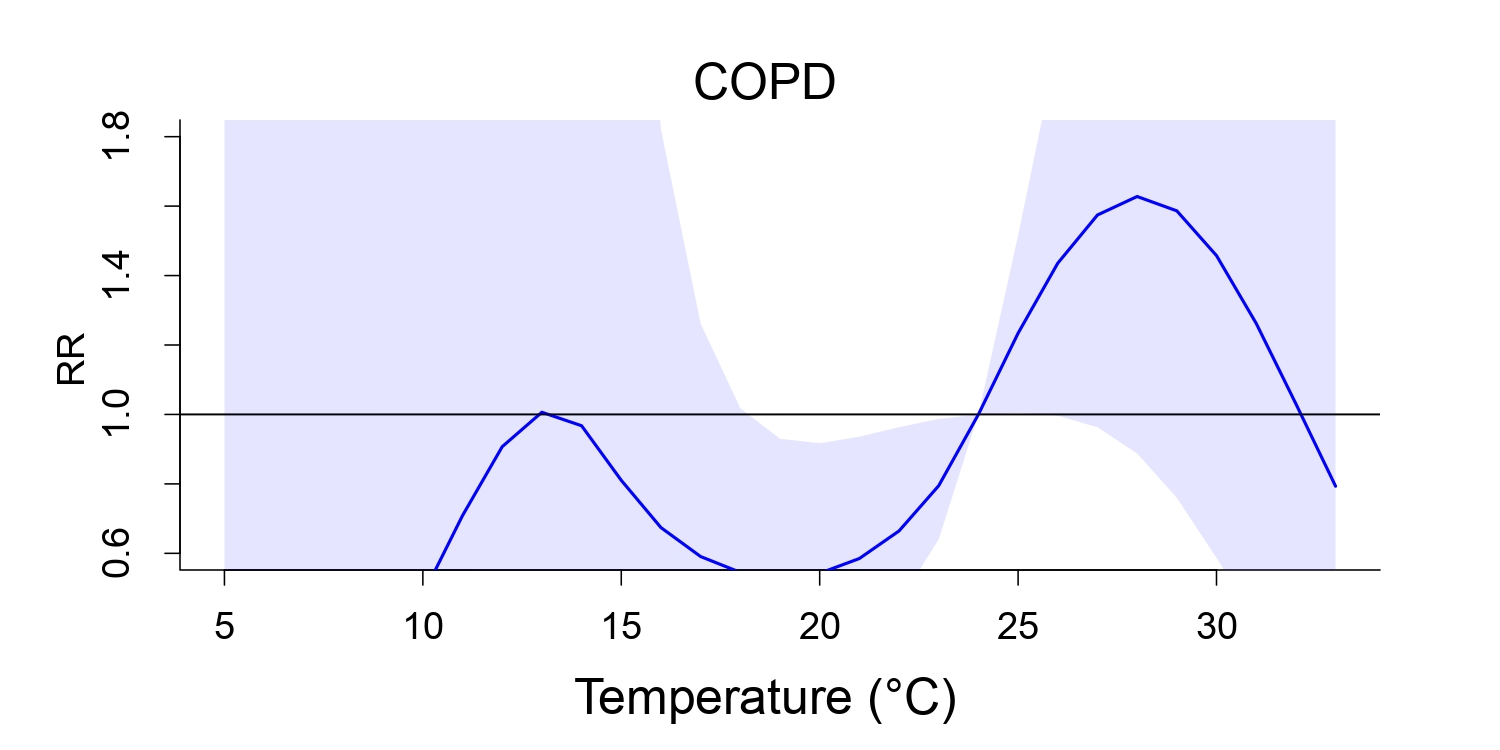


S4 Fig. Cumulative 6-day (lag 0-5) relative risk (95% confidence interval) of deaths from COPD in the elderly men associated with daily PM2.5 concentrations relative to Q1 levels (17 μg/m3) after adjusting for daily average temperature


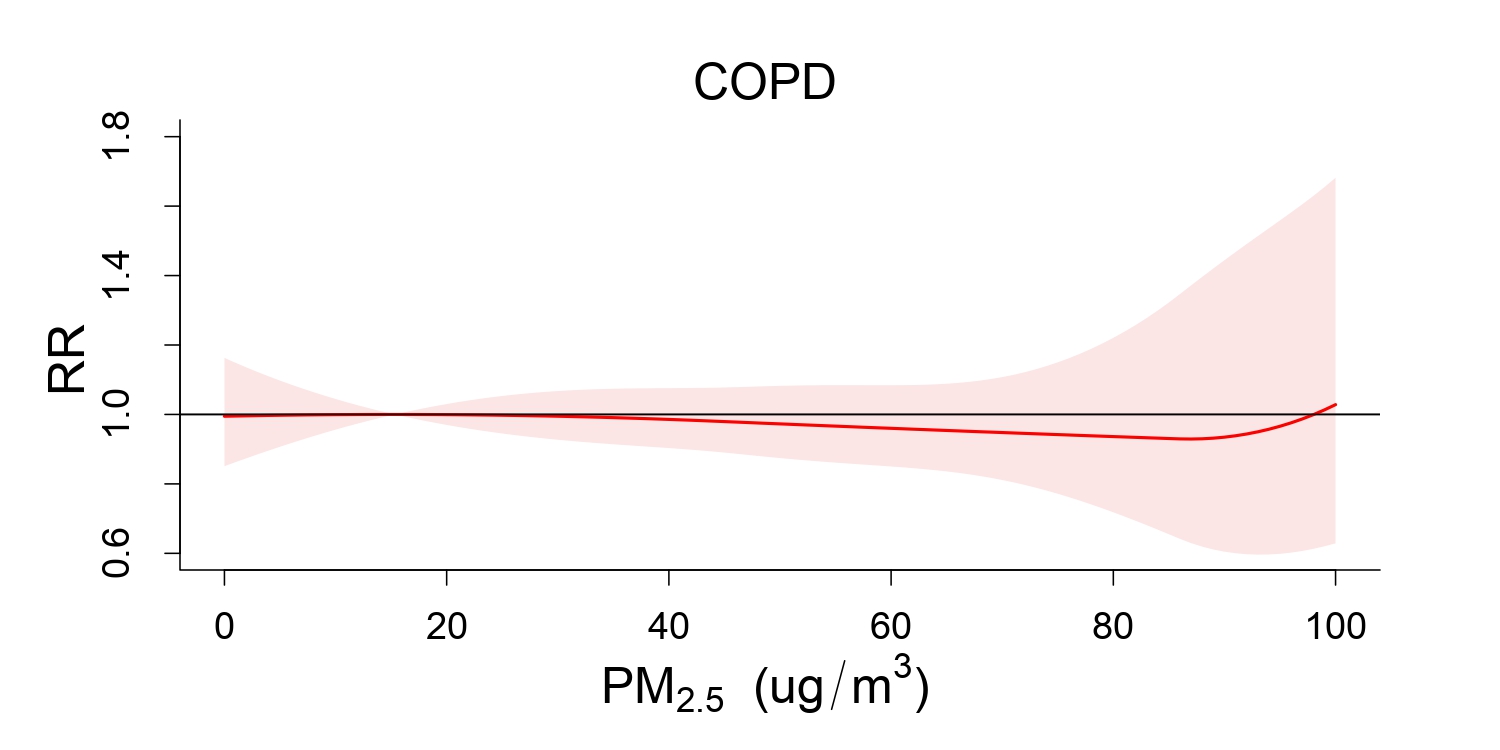


S5 Fig. Cumulative 6-day (lag 0-5) relative risk (95% confidence interval) of sex-age specific emergency room visits for asthma and COPD associated with daily PM2.5 levels relative to Q1 level (17 μg/m3) after adjusting for daily average temperature


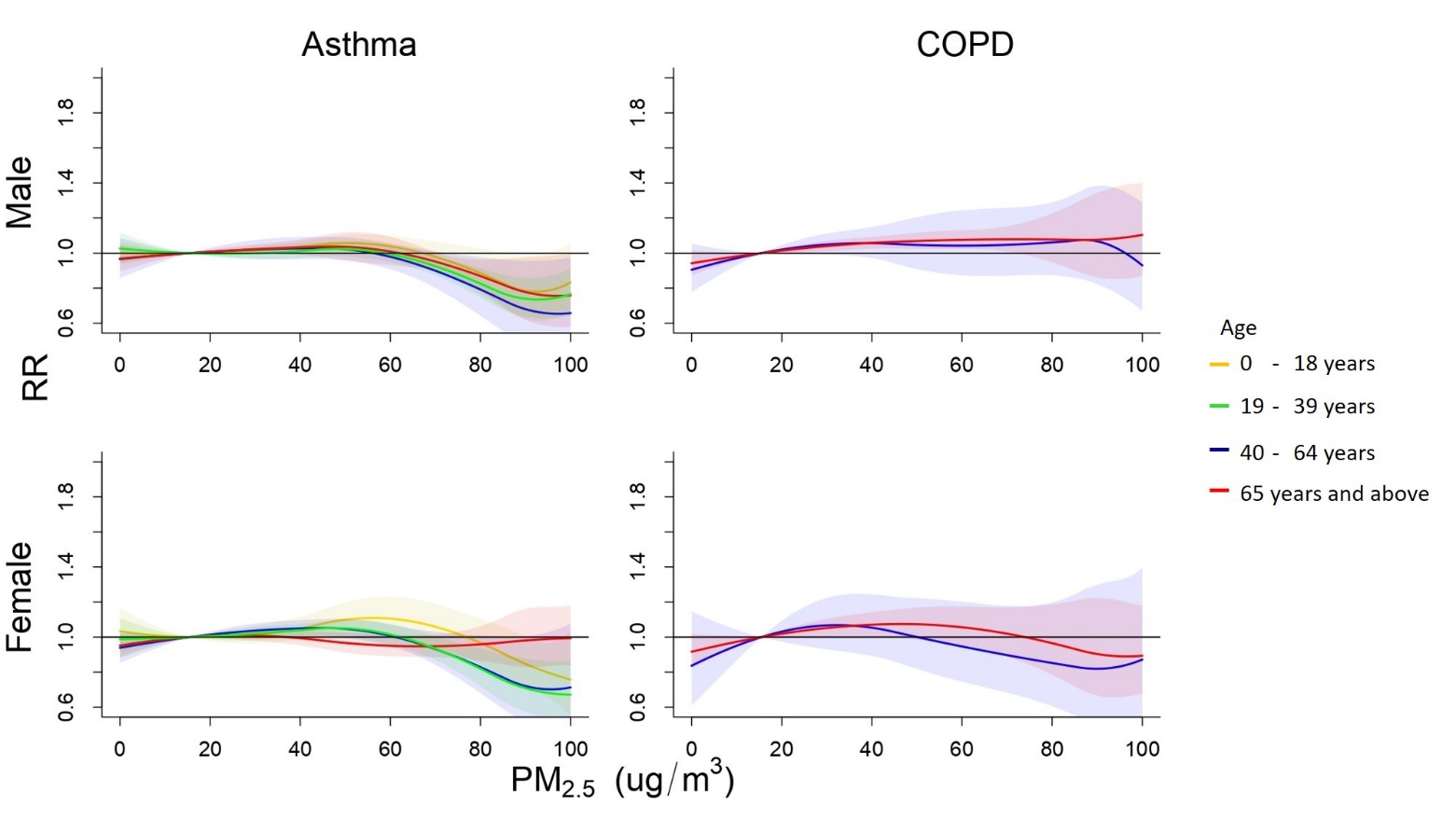


S6 Fig. Cumulative 6-day (lag 0-5) relative risk (95% confidence interval) of sex-specific deaths from asthma and COPD in the elderly associated with daily O3 levels relative to Q1 level (21 ppb) after adjusting for daily average temperature


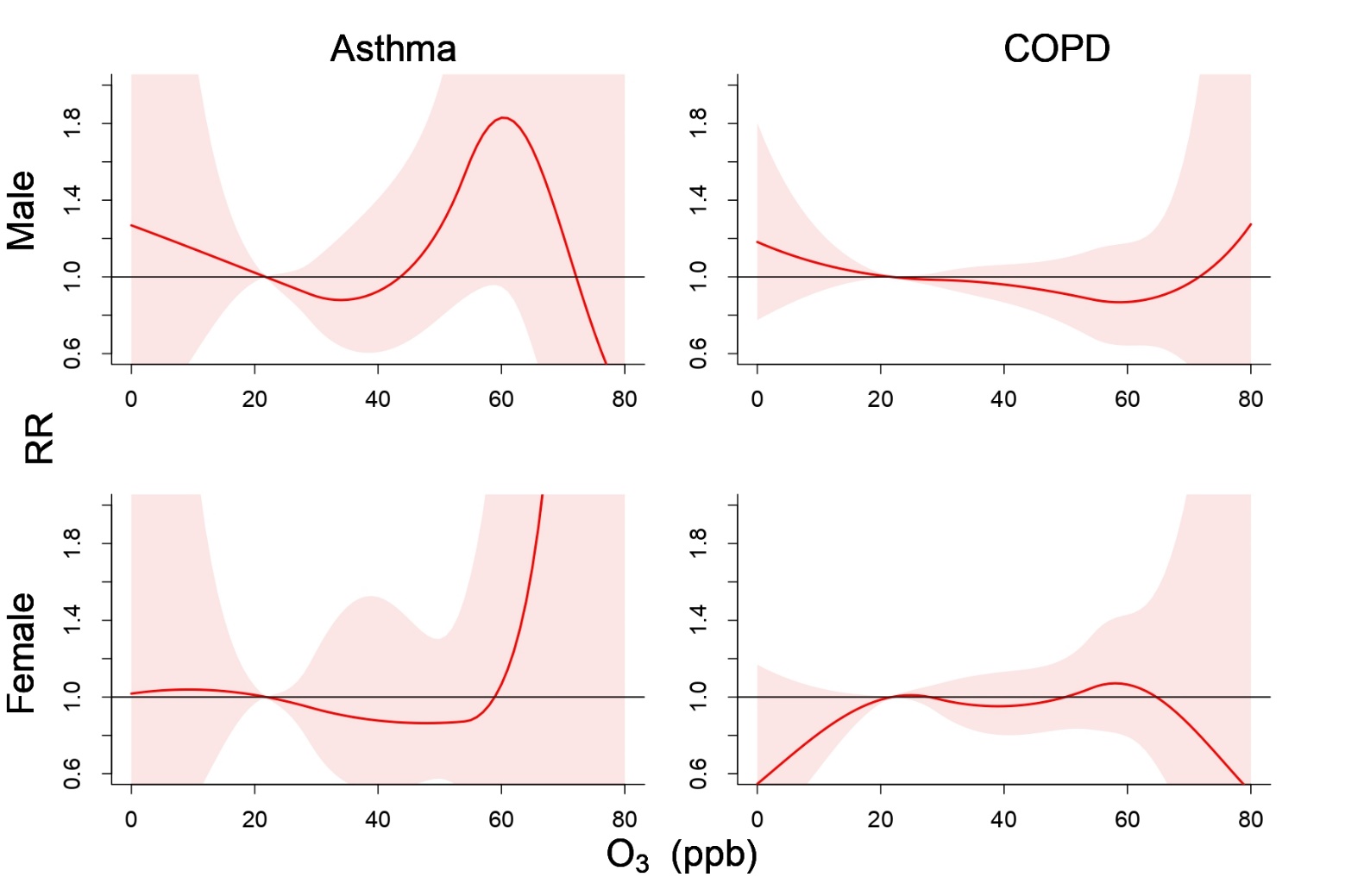


S7 Fig. Cumulative 6-day (lag 0-5) relative risk (95% confidence interval) of sex-age specific emergency room visits for asthma and COPD associated with daily O3 levels relative to Q1 level (21 ppb) after adjusting for daily average temperature


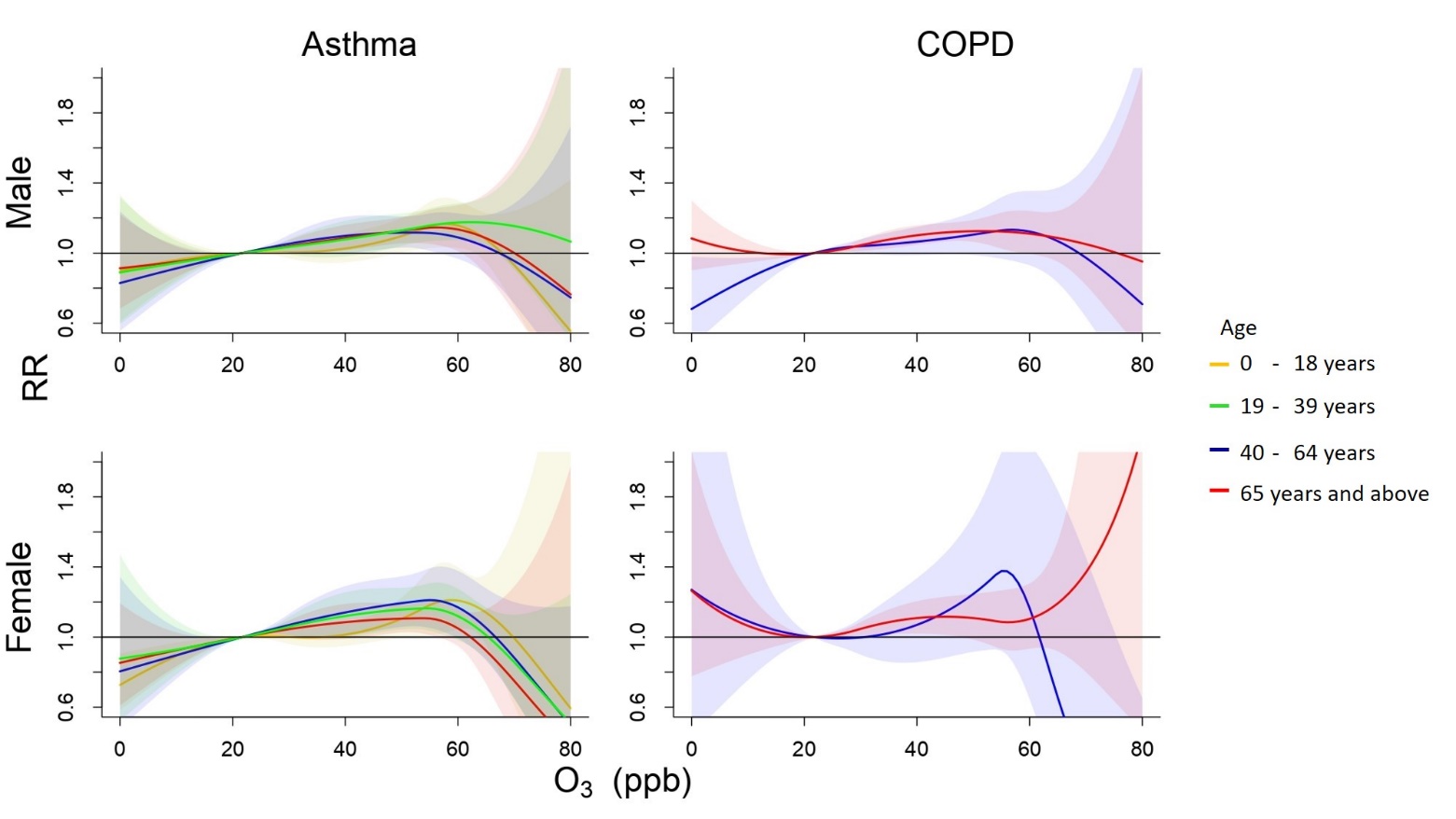

Supplement: S1 File — (DOCX) [file pone.0253814.s001.docx]
