# Supplementary material for: Mortality and morbidity of asthma and chronic obstructive pulmonary disease associated with ambient environment in metropolitans in Taiwan
Source: PLoS One. 2021 Jul 6;16(7):e0253814. doi: 10.1371/journal.pone.0253814 (PMC8259956; doi:10.1371/journal.pone.0253814)
Supplement: Attachment — Submitted filename: 0306 respone to reviewer PONE.docx [file pone.0253814.s003.docx]

Review Comments to the Author

Please use the space provided to explain your answers to the questions above. You may also include additional comments for the author, including concerns about dual publication, research ethics, or publication ethics. (Please upload your review as an attachment if it exceeds 20,000 characters)

Reviewer #1: 1. Authors would address that asthma definition included acute and chronic diagnosis in Discussion section.

Response: Thank you for your comment. We add some references in the discussion section to make it clearer. Please refer to line 236-244: ”Asthma is one of the most common chronic non-communicable diseases [1]. This disease has differential diagnosis between the acute and the chronic. Most acute asthma patient known without laboratory test or chest radiographs because the symptom of the patient easily diagnoses such as breathlessness, inability to speak more than sort phrases, use of accessory muscle, or drowsiness [2]. Otherwise, chronic asthma patients usually suffer from this chronic condition (long-lasting or recurrent) and the physical symptoms are not always present in asthma sufferers, and it is possible to have asthma without presenting any physical maladies during an examination [3]. So, chronic asthma patients should undergo several test such as spirometry test or bronchoprovocation test before they are diagnosed [4].”

1.	Shoraka H, Soodejani M, Abobakri O, Khanjani N. The Relation between Ambient Temperature and Asthma Exacerbation in Children: A Systematic Review. Journal of Lung Health and Disease. 2019;3(1):1-9.

2.	Fergeson JE, Patel SS, Lockey RF. Acute asthma, prognosis, and treatment. J Allergy Clin Immunol. 2017;139(2):438-47. Epub 2016/08/25. doi: 10.1016/j.jaci.2016.06.054. PubMed PMID: 27554811.

3.	Bousquet J, Bousquet PJ, Godard P, Daures JP. The public health implications of asthma. Bulletin of the World Health Organization. 2005;83(7):548-54. Epub 2005/09/24. PubMed PMID: 16175830; PubMed Central PMCID: PMCPMC2626301.

4.	Krishna Sailaja A. An overall review on chronic asthma. International Journal Pharmaceutics and Drug Analysis. 2014;2(3):275-9.

2. Generally, maximum daily mean of 8 hour averages of ozone concentration is known to affect health effects. This should be also addressed.

Response: Thank you for your comment. We already addressed that the analysis using 24-h average concentration of fine particulates matter (PM2.5) and ozone (O3) in the methodology section. Please refer to line 117-119:” We used 24-h average concentration of fine particulates matter with diameters of less than 2.5 micron (PM2.5) and ozone (O3) in this study. Refer to our previous study [5], 24-h average concentration of ozone is the preferred metric instead of 1-h maximum concentration and 8-h maximum concentration of ozone for assessing the association with outpatient visits for total respiratory diseases.”

5.	Lin Y-K, Chang S-C, Lin C, Chen Y-C, Wang Y-C. Comparing ozone metrics on associations with outpatient visits for respiratory diseases in Taipei Metropolitan area. Environmental Pollution. 2013;177:177-84. doi: <https://doi.org/10.1016/j.envpol.2012.12.010>.

Reviewer #2: Dear Authors,

All my comments and suggestions were addressed and in my opinion the manuscript is ready to be published now.
